# “Somebody was standing in my corner”: a mixed methods exploration of survivor, coach, and hospital staff perspectives and outcomes in an Australian cancer survivorship program

**DOI:** 10.1007/s00520-023-07908-y

**Published:** 2023-07-21

**Authors:** Tassia Kate Oswald, Leva Azadi, Sue Sinclair, Sharon Lawn, Paula Redpath, Liam Beecroft, Miles Ranogajec, Jeannie Yoo, Anthony Venning

**Affiliations:** 1 Discipline of Behavioural Health, College of Medicine and Public Health, Flinders University, Sturt Rd, Bedford, South Australia 5042 Australia; 2 Psychological Medicine, Institute of Psychiatry, Psychology & Neuroscience, King’s College London, London, SE5 8AB UK; 3 Remedy Healthcare Group, 271 Spring Street, Melbourne, VIC 3000 Australia; 4 Ramsay Health, Level 3, 5 Talavera Rd, Macquarie Park, New South Wales 2113 Australia; 5 Discipline of Public Health, College of Medicine and Public Health, Flinders University, Sturt Rd, Bedford, South Australia 5042 Australia

**Keywords:** Cancer survivorship, Care transition, Self-management, Health coaching, Low-intensity cognitive behavioural therapy, Mental health

## Abstract

**Purpose:**

Cancer survivorship in Australia continues to increase due to new methods for early detection and treatment. Cancer survivors face challenges in the survivorship phase and require ongoing support. A telephone-delivered cancer survivorship program (CSP), including health and mental health coaches, was developed, piloted, and evaluated in Eastern Australia.

**Methods:**

Cancer survivors’ (*n* = 7), coaches’ (*n* = 7), and hospital staff (*n* = 3) experiences of the CSP were explored through semi-structured interviews. Quantitative data routinely collected throughout the pilot of the CSP was described (*N* = 25).

**Results:**

Three syntheses and 11 themes were generated through thematic analysis. The first synthesis centred around operational factors and highlighted a need to streamline communication from the point of recruitment, through to program delivery, emphasising that the program could be beneficial when timed right and tailored correctly. The second synthesis indicated that the CSP focused on appropriate information, filled a gap in support, and met the needs of cancer survivors by empowering them. The third synthesis focussed on the value of mental health support in the CSP, but also highlighted challenges coaches faced in providing this support. Descriptive analysis of quantitative data indicated improvements in self-management, weekly physical activity, and meeting previously unmet needs.

**Conclusions:**

Cancer survivors expressed appreciation for the support they received through the CSP and, in line with other cancer survivorship research, predominantly valued just having somebody in their corner*.*

**Implications for cancer survivors:**

Recommendations are made for improving cancer survivorship programs in the future.

## Introduction

Cancer is a major cause of illness in Australia and there are currently over one million Australians who are either living with, or have lived with, cancer [1, 2]. Following a diagnosis, the active cancer treatment phase involves extensive contact with specialists and medical settings [3, 4], which has significant social, emotional, and economic impacts on individual patients, their families, and the wider community [5].

Between 1986 and 1990, approximately 50% of cancer patients survived for at least 5 years after their diagnosis, but recent figures indicate that this number has improved, rising to close to 70% of people between 2011 and 2015 [1]. By the end of 2014, almost half a million Australians were alive who had been diagnosed with cancer in the previous 5 years [1]. The proportion of individuals who have ever been diagnosed with cancer, and survived, continues to increase due to new methods being used for early detection and advancements in treatment technologies [6]. However, beyond the active treatment phase, those who have survived cancer often face ongoing physical, psychological, and financial challenges as a result of the disease, that require self-management and/or ongoing interventions [6]. Common physical and psychological experiences include pain, fatigue, sleep problems, memory and concentration difficulties, anxiety, and depression [7–10]. In particular, young adult cancer survivors may experience unique challenges related to their careers, intimate relationships, and sexual and fertility issues [11]. These challenges can be interrelated or occur simultaneously for some people, which compounds the management required [1] and highlights a critical need for a continuum of care from active cancer treatment into the survivorship and self-management phase [12].

Qualitative research by Lubberding and colleagues [13] found that cancer survivors report feeling unprepared for the post-treatment phase of their cancer journey and that their ongoing symptoms often remain unknown to health care providers or are not prioritised. Cancer survivors have commented on experiencing suboptimal referral processes into continuing supportive care services [13, 14]. Some survivorship interventions in the UK and USA have indicated the potential benefits of supporting cancer survivors in the post-treatment phase, including improved reported health outcomes and fewer social limitations [12], as well as increased access to support pathways and services [15, 16]. In Australia, recommendations for cancer survivorship care include a systematic, multidisciplinary care approach that optimises self-management and coordination, and stratifies services to meet patient need [17]. More recent international reviews suggest that telemedicine interventions can be effective for addressing the physical and psychosocial needs of cancer survivors [18, 19]. However, not much is known about the perspectives and effectiveness of survivorship planning for cancer survivors in Australia.

### The current study

The current study aims to fill existing gaps in evidence and practice standards around what constitutes an effective and accepted cancer survivorship care program [12]. To do this, a telephone-delivered cancer survivorship program (CSP) was developed and piloted in Eastern Australia. Using a mixed methods approach, the current study aimed to (1) explore cancer survivors’, coaches’, and hospital staff *perspectives* of the CSP through a *qualitative* exploratory research design, and (2) describe cancer survivor *outcomes* using relevant *quantitative* data routinely collected throughout the CSP.

## Methods

Ethical approval to conduct this study was gained from the Flinders University Human Research Ethics Committee (Research Project #3979) and relevant Ramsay Healthcare Australia hospital governance was also obtained.

### The Ramsay Connect Cancer Survivorship Program (CSP)

The Australian healthcare system is a mixed model, including both public government-funded universal coverage of various medical costs, along with coverage via private health insurance or self-funding [20, 21]. In 2013, a nationally representative survey indicated that 58.4% of Australians with a current cancer diagnosis were covered by private health insurance [22].

The Ramsay Connect Cancer Survivorship Program (CSP) was developed by Ramsay Healthcare Australia and Remedy Healthcare (through a joint venture partnership, *Ramsay Connect*) to address the unmet needs of cancer survivors when they complete hospital-based cancer treatment. Ramsay Healthcare Australia is the largest private health care provider of cancer care in Australia, and Remedy Healthcare is a national provider of home-based and virtual nursing, allied health, and evidence-based self-management, mental health, and health coaching services. The CSP was piloted with cancer survivors in the private healthcare system at no additional cost to cancer survivors or private health insurance funders.

The CSP pilot was undertaken at three Ramsay Hospital sites in Australia. At each hospital, a site champion was identified to lead engagement prior to commencement and throughout the pilot. Once the pilot was launched, an additional engagement session was delivered to the Director of Clinical Services at each hospital to encourage ongoing engagement in the CSP.

The CSP involved three main components, which are described in Table 1. The first component was support from nurses and cancer care navigation from a Cancer Care Navigator (experienced cancer nurse) during treatment in the hospital setting. The other main component of the CSP was an adapted version of a health coaching program called “HealthierMe”, which was tailored to include relevant information for cancer survivors following treatment. The final component of the CSP was a low-intensity cognitive behavioural therapy program called “MindStep”. Overall, the CSP aimed to maintain continuity of care in the transition from hospital- to community-based care. Cancer survivors who were identified as having unmet needs at the end of their hospital-based treatment were provided with a health coach, and a mental health coach in cases where cancer survivors screened positive for symptoms of depression and anxiety. The program was designed to enable strong links between health coaches and a cancer survivor’s hospital-based cancer team, along with other linkages to their GP, and community-based care options, to support and empower them to move towards self-management of their cancer-related and other health issues in the community context. The CSP was underpinned by a *theory of change* and was expected to result in participants gaining self-management skills, improved connection with services, improved self-care, and reducing any potential unmet needs in their cancer survivorship phase.

**Table 1:** Description of the Ramsay Connect Cancer Survivorship Program (CSP), incorporating HealthierMe and MindStep

Component	Description and content	Providers
*Nursing support and cancer care navigation* In the hospital setting	Patients were provided with nursing support and cancer care navigation in the hospital setting. Towards the end of their treatment in the hospital setting, a risk and screening assessment survey was completed to identify any unmet needs of cancer survivors, and their potential eligibility for the *HealthierMe* component of the CSP (described below).	Nurses were responsible for supporting patients with treatment in the Ramsay hospital setting. Cancer Care Navigators were experienced cancer nurses who provided healthcare navigation support to patients in the hospital-setting and championed the CSP in their hospital. Both nurses and Cancer Care Navigators (whoever was available) had the responsibility of organising the risk and screening assessment surveys. These surveys were completed either by the cancer survivor alone, or together with a nurse or Cancer Care Navigator.
*HealthierMe* Telephone-delivered	*HealthierMe* was the core component of the CSP and was suitable for all cancer survivors who had a need for self-management support. HealthierMe consists of telephone-based health coaching, self-management support, and care coordination. The standard program was adapted for this cohort of cancer survivors to include core modules relating to managing general emotional wellbeing, as well as anxiety and depression; managing lifestyle factors including diet, sleep, smoking, and especially physical activity; health services navigation; management of treatment side effects and symptoms; management of medicines; and supporting family and social life.	*HealthierMe* is delivered by Health Coaches at Remedy Healthcare who are primarily qualified as nutritionists and have extensive experience in chronic condition self-management, using behavioural change and motivational interviewing techniques. Training is provided to Health Coaches by *Health Change Australia*. Health Coaches who were involved in this pilot received additional training to prepare them to work with a cohort of cancer survivors specifically. This included multiple training sessions covering specific cancer-related content and participating in a webinar on cancer survivorship run by the *Peter MacCallum Institute.*
*MindStep* Telephone-delivered	*MindStep* is a telephone-based low-intensity cognitive behavioural therapy program, which has been shown to help people who are experiencing depression and/or anxiety [23]. The MindStep program was offered to cancer survivors who were identified by their Health Coach as experiencing mild-to-moderate psychological distress (i.e. had a score of 3 or greater on one or both sub-scores of the Patient Health Questionnaire-4).	*MindStep* is delivered by MindStep Coaches at Remedy Healthcare, who are trained in a range of techniques such as behavioural activation, behavioural experiments, worry management, psychoeducation, goal setting, and problem solving (see Lawn et al. [24] for more details). In preparation for the CSP, MindStep coaches also viewed the webinar on cancer survivorship run by the *Peter MacCallum Institute.*

#### Screening and eligibility

The screening process for participant eligibility included a preliminary risk screen as well as an assessment of unmet needs. The preliminary risk screen identified cancer survivors who would most benefit from the program, including those who had long or complex treatment pathways, lived in regional or remote locations, had lower levels of activation and health literacy, had polypharmacy and/or co-morbidities, lived alone or had a limited informal support network, and/or did not have established support services in the community.

Cancer survivors who screened positive for one or more of these risk screening criteria were also assessed for unmet needs across domains of information, financial, access and continuity of care, and coping, sharing and emotion needs, using the short form Survivor Unmet Needs measure (SF-SUNS, described in ‘Survivor unmet needs and patient activation measures’). Cancer survivors who were found to have moderate, high, or very high unmet needs (indicated by a domain sub-score of > 1.5 on the SF-SUNS) in any of the domains were offered the CSP.

Cancer survivors were not eligible if they were continuing active treatment, were experiencing cognitive impairment, were < 18 years old, or were highly activated individuals (e.g. had low levels of unmet needs). It was anticipated that the CSP would not be beneficial for cancer survivors who were highly connected with their required supports and had low levels of unmet needs.

### Qualitative component

A qualitative exploratory research design was used to explore cancer survivors’, coaches’, and hospital staff *perspectives* of the CSP. Details of the qualitative component of the research are detailed in the following subsections ‘Participants’, ‘Procedure’, and ‘Analysis’.

#### Participants

Four types of participants were invited to participate in the qualitative aspect of the research project via email. Individuals recruited included (1) cancer survivors, (2) hospital staff (e.g. nurses, Cancer Care Navigators, clinical directors, or nursing unit managers who were involved in either program promotion, recruitment, or operational planning), (3) Health Coaches, or (4) MindStep Coaches. A total of 35 individuals were contacted for interviews (three health coaches, four MindStep coaches, 10 hospital staff, and 18 cancer survivors). Email addresses were available to the Flinders University researchers through the collaborating partners. Individuals who wanted to participate in the study responded to the email invitation and provided written consent. Participants in the qualitative component of the research received a $50 (AUD) gift card for their time.

#### Procedure

One-to-one semi-structured interviews were conducted with participants, either over the phone or via *Microsoft Teams*, by one researcher (TKO). Interview guides, specific to each type of participant, comprising of open-ended questions and prompts were used. Participants were asked questions pertaining to their experiences in the CSP (e.g. “How would you describe your experience of the program and why?”), outcomes achieved through the CSP (e.g. “What outcomes were achieved by your clients?”), how the CSP was integrated into existing care (e.g. “From your point-of-view, how do you think the program was integrated into the wider care team?”), and what participants believed did and did not work in the CSP. At the point of the interviews, cancer survivors had completed the CSP, health coaches and MindStep coaches had been working in the CSP for varying lengths of time, and all hospital staff had been involved since inception of the CSP.

#### Analysis

All interviews were audio recorded, de-identified, and transcribed. Thematic analysis of the data was undertaken as described by Braun and Clarke [25], involving six phases: (1) in-depth familiarisation with the data, through listening to interview recordings and re-reading transcripts; (2) generating initial codes by identifying interesting features across the dataset in a systematic way; (3) searching for themes by collating relevant codes; (4) reviewing the generated themes by generating a thematic map of the analysis; (5) defining and naming the themes, alongside refinement of the thematic map; and (6) writing up the analysis into an interpretable piece with extract examples that relate to the themes. Two researchers (TKO and AV) discussed the codes and themes, and example quotes relating to the themes were provided to co-authors.

### Quantitative component

Quantitative data routinely collected throughout the CSP was used to describe cancer survivor outcomes. Details of the quantitative component of the research are detailed in the following subsections ‘Participants’, ‘Procedure’, and ‘Analysis’.

#### Participants

Retrospective data were drawn from 25 cancer survivors who had completed the CSP between September 2020 and December 2021. At the time of this research 38 cancer survivors had commenced the CSP, but several were still progressing through the program. This sample (*n* = 25) represents 100% of all cancer survivors who had completed the program in this time period. Data for program completers were only used if the individuals had provided consent for their data to be used for research purposes at first contact.

#### Procedure

De-identified data were drawn from *SalesForce* at Remedy Healthcare. SalesForce is a web-based system used by Remedy Healthcare to document activity and service outcomes, as well as provide a mechanism for clinical supervision [23]. The data extracted from SalesForce are described below.

##### Sociodemographic variables

Sex, age, and cancer diagnosis of participating cancer survivors were extracted from SalesForce. The number of telephone calls and days the cancer survivor remained in the CSP were also obtained.

##### Survivor unmet needs and patient activation measures 

Survivor unmet needs and patient activation were measured at baseline and completion of the CSP using the short form Survivor Unmet Needs (SF-SUNS) measure and the Patient Activation Measure (PAM). These baseline and post-program scores were extracted from SalesForce.

The SF-SUNS is a 30-item standardised psychometric measure of unmet needs for cancer survivors, which has shown strong reliability and validity [26]. The SF-SUNS assesses cancer survivors’ unmet needs over the last month across four domains: *information* (3 items)*, financial* (7 items), *access and continuity of care* (5 items), and *coping, sharing and emotional needs* (13 items). Twenty-eight items were used and response options range from 0 (*no unmet need*) to 4 (*very high unmet need*). Each domain is scored separately, with the total score divided by the number of items in that domain. If item scores are missing, the number divided into the total score is reduced. A score under 1.5 indicates no-to-low unmet needs, 1.5 to 3 indicates moderate unmet needs, and > 3 indicates high-to-very high unmet needs.

The PAM is a 10-item measure which is used to assess levels of patient “activation”; an individual’s understanding, competence, and willingness to participate in and self-manage their health as required [27–29]. Respondents are asked to respond to questions (e.g. “I know how to prevent further problems with my health condition”) on a scale ranging from disagree strongly to agree strongly. PAM scores range from 0 to 100, with higher scores indicating a more highly “activated” individual. The PAM has good psychometric properties (valid and reliable) and can be used to assess a person’s underlying knowledge, skills, and confidence to manage their own healthcare [27].

##### Physical activity 

Self-reported cancer survivor physical activity was obtained from SalesForce at baseline and post-program. Physical activity was presented as the number of physically active minutes reported per week.

#### Analysis

Descriptive analysis of the routinely collected quantitative data from the CSP pilot was conducted to explore cancer survivor outcomes on relevant measures. Means and standard deviations (SD) were calculated and presented for each outcome pre- and post- the CSP.

## Results

### Qualitative results

**Fig. 1:**
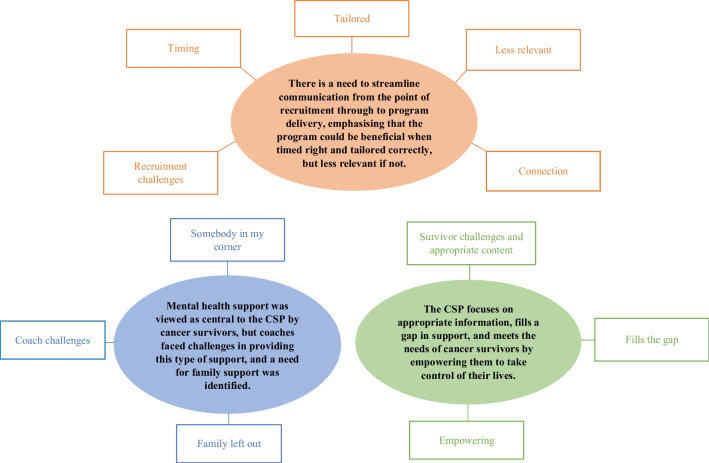
Map of syntheses and themes generated though thematic analysis (CSP = Cancer Survivorship Program)

Seventeen participants were recruited for interviews (49% of all individuals approached): seven cancer survivors (71% female); three hospital staff members (66% female); three health coaches (66% female); and four MindStep coaches (100% female). Due to the small number of hospital staff recruited, we have not specified their roles, to maintain anonymity. Interviews went for an average of 39 minutes (range: 15–100 minutes). Three syntheses and 11 themes were generated. Figure 1 presents a map of the thematic analysis. Accompanying qualifiers are used in the results to signify the participant for a quote (cancer survivor (CS); hospital staff member (HS); health coach (HC); and MindStep coach (MSC)).

#### Synthesis 1

The first synthesis centred around operational factors and highlighted a need to streamline communication from the point of recruitment through to program delivery, emphasising that the CSP could be beneficial for cancer survivors when timed right and tailored correctly, but less relevant if not. Synthesis 1 included five themes: (a) *recruitment challenges,* (b) *timing*, (c) *tailored*, (d) *less relevant*, and (e) *connection*.

##### Recruitment challenges

Hospital staff expressed facing several challenges when recruiting and referring cancer survivors into the CSP. Foremost, hospital staff expressed difficulties in identifying cancer survivors who met the eligibility criteria for the CSP, with one participant commenting “I was struggling to find anybody who was fitting the original criteria of you know finishing treatment…. there’s fewer treatment regimes where there’s a definite end date” (HS1). While the CSP was designed for cancer survivors who had ended their active cancer treatment, the eligibility criteria for referral into the program changed part-way through the pilot due to this difficulty in identifying individuals who matched the original criteria. The program was consequently opened by Ramsay Healthcare Australia to cancer survivors who were still in longer-term or maintenance treatment, although this expansion of the eligibility criteria was not clearly defined and potential consequences of this were not considered at the time.

Other practical constraints surrounding recruitment in the clinical setting were also raised. For example, a hospital staff participant indicated “I think the paperwork side of it was a stumbling block for lots of the nurses because in oncology some patients are only there for an hour or so and you’ve only got just enough time to do the clinical side of it, rather than adding extra things in” (HS2), while another participant indicated that “there was an unrealistic expectation…the real challenges of what was happening in the actual clinical environment weren’t considered” (HS3). Some hospital staff also found the screening tools time-consuming and complex, which meant that screening practices across hospitals and between staff members varied considerably. One hospital staff member remarked, “I kind of did my thing my way in certain respects, so there may have been some clinician-based bias about the people who got referred in the end, rather than everybody gets an assessment and a score” (HS1). This had implications for which cancer survivors were referred into the program and consequently affected survivor and coach experiences.

There were also a range of challenges and successes around promoting the program to cancer survivors. In particular, introducing the concept to cancer survivors towards the end of their treatment journey made it difficult to get them on board, “it was a bit hard to engage them and understand what the benefits might be for them because by then they were already thinking I’m nearly finished” (HS1). Hospital staff voiced the importance of early integration of the program into conversations with patients. Cancer survivors themselves also indicated that it would be good “if a communication could start right away when you go and see these guys” (CS1). Hospital staff also mentioned the important role of doctors when introducing the program to cancer survivors, “the older men sometimes think that their doctors just know more because they’re doctors…the doctor tells him to do it, he’ll do it…if a nurse tells him it’s more of a guideline; you don’t need to do it” (HS2), and discussed the possibility of removing eligibility barriers all together by making the program “part of the standard practice of care” (HS2) and “just part of what we do” (HS1).

The way the program was described to cancer survivors in the hospital setting also had flow-on effects to the health coaches when they made initial contact with cancer survivors to start the program over the phone. Where cancer survivors were clear about the purpose of the program, it was easier for the health coaches to commence; “Some hospitals were better than others in that process…you’d call the client and they’d be like ‘yeah …. spoke about this, this is great, yeah I know all about it.’ And then some were like ‘sorry, who are you, and what is this?’ and you kind of were on the back foot early and then kind of had to sell the program to them as well” (HC2).

##### Timing

Following changes in the CSP eligibility criteria that allowed cancer survivors in ongoing long-term treatment to be admitted to the program, the timing of the CSP in a cancer patient’s journey emerged as a central point of discussion. For individuals who were terminally ill and receiving ongoing maintenance treatment for their cancer, the support offered by the program was seen as too late in their journey. One individual reported “to me it wasn’t happening at the time it was supposed to happen” (CS2). They felt it would have been helpful at the time they were first diagnosed as that was when their life changed, “not this far down the track and with what I’d been dealing with over the last six years” (CS2). A health coach added to this when reflecting on their experience working with a person in ongoing cancer treatment, “[Patient] is a little bit less receptive to some of the things I have to offer because [they] said ‘I have been going through this forever’” (HC1).

Multiple cancer survivors expressed the need for support from the beginning of the cancer diagnosis and commencement of treatment (e.g. “I would have benefitted from it at the start of my diagnosis as well” (CS3))*,* but they also acknowledged that it may have been too much at this time; “I may not have had the time to have taken the phone calls…because it’s actually a really busy period” (CS3), and “there was so much going on. I think it might have been a layer too much” (CS5). This view was also shared by both hospital staff, “while they’re on treatment their focus is getting through treatment and then getting well at the end of it…they were ready to receive information instead of being information overloaded during their chemo” (HS2), and MindStep coaches, “an ideal time would be a little bit after that stage [diagnosis], …because they can get more activated and can engage in some goals a bit more easily” (MSC4).

##### Tailored

There was a general consensus that the CSP could work for individuals at various stages of their cancer journey, but that it must be tailored appropriately when doing so, with clear “expectations and parameters” (MSC2). A health coach noted that “it could be really beneficial for people still going through treatment…[but] there needs to be work on that and adapt that a little bit” (HC1). In particular, hospital staff highlighted that cancer survivors with long-term, ongoing treatment have “a whole other series of things that they have to deal with” (HS1) and “their survivorship plans are a lot different than someone that’s having it as an early curative treatment” (HS2). In these cases, a shift in focus to “living with the cancer rather than what comes after it” (MSC2) was viewed as essential.

##### Less relevant

Given the program was specifically designed for individuals transitioning out of the cancer treatment phase, it was considered inappropriate for, and by, some individuals who did not fit the original eligibility criteria. For example, individuals undergoing maintenance treatment for terminal cancer who were referred into the program viewed some of the content and processes as inappropriate, “you can’t answer these questionnaires honestly, because they’re so irrelevant…‘in the past two weeks were you unhappy?’ … of course I’m unhappy” (CS2). Health coaches also echoed difficulties around working with cancer survivors who did not fit the original criteria, “it’s harder to kind of follow through with the program when they’re really just focused on ‘I have surgery next week’” (HC1). Some coaches voiced that the strategies were not realistic or effective as “they were doing the chemo or some sort of treatment, because they were terminal” (MSC3).

Similarly, when program screening was not systematic or consistent, it meant that some individuals were referred into the program despite possibly not meeting the needs criteria for admission. For example, a cancer survivor reported “I had really good support networks…I think for me my life pretty much went back to normal” (CS5), while one of the hospital staff participants indicated, “some of them [cancer survivors] I think thought they were helping me out…they thought they’d you know, they were helping me out by saying yes” (HS2), and that their experience transitioning back home was unlikely to have been different without the CSP.

##### Connection

A key feature of the CSP was to maintain continuity of care in the transition from acute cancer care in hospital to community-based cancer care by providing a health coach with strong links to their hospital-based cancer team and GP. While “good rapport” (HS1) was built between hospital cancer care navigators and health coaches in the referral process, connection between members of the broader multidisciplinary care team was seen to be lacking across the program. Health coaches indicated that “the multi-disciplinary sort of like team approach isn’t quite there” (HC3) and that “there’s no correspondence or any other real integration” (HC2). Hospital staff also voiced a lack of feedback, “a lot of the time I feel like we’re pushing outwards, we were making referrals out to here…then you’re not sure what’s happened” (HS1).

While some participants highlighted the benefits that care team connection would offer, “so we can all kind of be working towards the same thing” (HC1), most did not think that it would be practical or add much value. A cancer survivor commented, “I don’t know how much information they could pass on that would be helpful that I couldn’t pass on myself” (CS3), while a health coach added “from a time-consuming point of view…it would be more work that may not give that much” (HC2). Issues around time management and patient confidentiality were raised as barriers and, overall, both cancer survivors and health coaches believed that cancer survivors could share important information or updates (e.g., around upcoming appointments and results) themselves. Hospital staff expressed a desire for “closing that loop with some feedback…[and] want[ing] to know how those guys [cancer survivors] got on” (HS1) in the CSP, highlighting the importance of feedback from health coaches to hospital staff. While an “after treatment care plan” (survivorship plan) was originally intended to facilitate connection between the care team, most participants did not recall seeing or using the plan beyond initial referral to the health coach. Similarly, while a summary report was intended to be sent to the GP upon completion of the program, this often did not transpire.

#### Synthesis 2

The second synthesis indicated that the CSP focuses on appropriate information, fills a gap in support, and meets the needs of cancer survivors by empowering them to take control of their lives and build self-confidence. Synthesis 2 included three themes: (a) *survivor challenges and appropriate content*, (b) *fills the gap*, and (c) *empowering*.

##### Survivor challenges and appropriate content

Both cancer survivors and clinicians highlighted a number of challenges that survivors face in the post-treatment phase, which aligned with the focus of the CSP. Recurring survivor challenges discussed included *weight loss/gain* (noted by two HCs, one MSC, and one CS), *sleep* (noted by one HC, one HS, one MSC, and three CSs), *loss of strength, fitness and exercise* (noted by two HCs, two HSs, one MSC, and five CSs), *fatigue* (noted by two HCs, one HS, one MSC, and three CSs), *mental health problems* (noted by three HCs, two HSs, and four CSs), and *fear of cancer reoccurrence* (noted by three MSCs and one CS). A MindStep coach remarked that, “it’s not just about the cancer…there’s multiple things happening…their world is changing…their view of themselves is changing…their future [is] changing, but the rest of the people around them, everything stands still” (MSC2). The culmination of these cancer-related challenges, alongside other regular life demands, was described by cancer survivors as “so overwhelming” (CS6), with health coaches noting that survivors “just wanted to get back to their normal lives” (HC3).

The CSP content and format were seen to meet the needs of cancer survivors; “there’s been no gaping holes… I don’t think there is anything that I have thought ‘oh we should talk about this’ that hasn’t been in a module” (HC1). The modules included in the program encompassed important topics which guided the phone calls and were self-directed by survivors to ensure relevance. One health coach reported “giving them that kind of menu of options was good… letting them know that this is what we can cover, what is a priority to you…you wanted to keep it relevant to them” (HC2). A hospital staff member also commented “the online [telephone] stuff was easy to sell to patients as well because they didn’t have to turn up for an appointment” (HS2).

##### Fills the gap

A distinct cessation was felt in the relationship between the acute clinical care team and the cancer survivor once treatment was finished. This gap was acknowledged by hospital staff (“there’s a distinct end to the relationship between you know the care and the patient…as a service provider we weren’t really even acknowledging that they might need support further afterwards” (HS1)), health coaches (“they had so much contact with people at the hospital during the treatment and now not much at all” (HC1)), and cancer survivors themselves (“you are sort of not having regular contact” (CS6)). The role of the CSP was seen to successfully fill this gap and loss of support, and “a lot of them [cancer survivors] saw this as their kind of ongoing support network” (HS2). One cancer survivor reflected, “there’s kind of little gaps, and that’s when [the health coach] kind of stepped in to help me process what’s happened now that I have slowed down a little bit and to what I was faced ahead of me” (CS3). Another cancer survivor commented, “knowing that somebody was available for those regular check-ins certainly helped…with the transition between treatment and being back to normal, because I’m nowhere near normal yet” (CS6). Overall, survivors commented, “I have really appreciated that this help was available…I had a really positive experience through a really crappy time” (CS5), “when my particular type of cancer comes back again…it’s certainly something that I would go into very willingly and it could help at that time” (CS6), and “it was something that I would recommend to anyone who is going through cancer treatment” (CS3).

##### Empowering

Both the hospital staff and coaches discussed how the program was, “all about empowering them [cancer survivors]” (HC3) to navigate their health services and lifestyle management. A hospital staff member commented “we tell them what to do…it’s good advice but it’s not enough advice to empower the person to manage it themselves…but now we’re trying to get them to see the people they should before all those things start to happen so that maybe they’ve got much better tools to be able to cope with it themselves” (HS1). While some cancer survivors expected more direction from the program, the guidance offered by the health coaches was still viewed as beneficial, “I probably expected that I would have a little bit more guidance…a little bit prescriptive perhaps, whereas it was more like guiding, me taking responsibility and guiding it with a bit of reinforcement from [the health coach], which also worked for me” (CS5). Building self-confidence in cancer survivors was key to helping them transition back to independence at home. One survivor reflected, “I was frightened that I could have a fall or something you know, and it’s given me that confidence back now to just walk like normal” (CS1).

#### Synthesis 3

The third synthesis focussed on the value placed on mental health support by cancer survivors going through the CSP and highlights the challenges coaches faced in providing this type of support, as well as a need for emotional support for families. The synthesis included three themes: (a) *somebody in my corner*, (b) *coach challenges*, and (c) *gaps in family support.*

##### Somebody in my corner

Mental health difficulties were prominent in cancer survivors’ journeys and having emotional support was viewed as central to the CSP. Hospital staff commented that “they [cancer survivors] would go into the program to get mental health support” (HS2), and that they were “resistant to seeing a psychologist because of the cost” (HS2) or because “there is still a stigma attached” (HS1). One survivor commented, “I went to a psychologist… and I just thought she didn’t really have the knowledge or experience of a cancer patient… but as soon as I spoke to these guys, they were really prepared and had a pretty good understanding of what to expect from me and my experience” (CS3).

Cancer survivors reflected that “just talking, psychologically had a positive impact” (CS7) and that “it was just good to know that somebody was standing in my corner” (CS6). A number of cancer survivors also commented on the benefits of having a third-party person, “away from my family” (CS1), to turn to and problem-solve with.

##### Coach challenges

The health coaches, whose training and work predominantly centres around supporting patients with physical chronic conditions (e.g. diabetes), discussed feeling challenged and unprepared to have discussions about mental health with cancer survivors, despite receiving additional cancer-specific training. For example, a health coach remarked “I don’t want to say the wrong thing, I’m not sure how to support them in this scenario as well…it’s not a cohort we’re well-versed in having these deeper more health concerning conversations” (HC2), further expressing desires for more upskilling. While the option to refer cancer survivors onto the MindStep program for mental health concerns was available, another health coach remarked that survivors “appreciated that bond and the relationship that we’ve established …. happy to just keep it between us…and yeah, [cancer survivor] has declined MindStep” (HC3).

For cancer survivors who did take up the MindStep program, they had diverse views on what the program could offer for their mental health. Views ranged from “my mental coach was actually amazing. Her skills and knowledge were just – they were actually so impressive, the way she helped me” (CS3), to comments about the program being too “generic” (CS2) or “scripted” (CS6). The MindStep coaches reflected how their typical program delivery did need to be adjusted for this cohort of cancer survivors, who were facing “more complexity” (MSC2) compared to their typical participants. MindStep coaches found that they had to use “different language” (MSC4) and be “a bit flexible in how [they] use[d] the treatment type sometimes” (MSC4). The key difference from usual practice being “we really need to just let these people talk about their journey… just have that really open-ended questioning and just hear them out…rather than us just being like, ‘right, I just want to get to, you know, what’s the problem?’” (MSC1).

Both health and MindStep coaches also voiced difficulties around dealing with serious acute health incidents experienced by cancer survivors. Immediate concerns around PET scans or cancer reoccurrence, for example, affected delivery of the program and required different approaches. They also reported feeling as though their cancer knowledge could have been better, particularly around cancer types and treatment regimes, “it’s just like oh my goodness, there’s so much information there and you just don’t know what to say…and I’m like oh man this is so embarrassing…I just don’t understand it enough…it’s very confusing…that is the part where I’m just like all of this is hard” (HC3). This was especially important given the CSP was eventually offered to cancer survivors in ongoing, longer-term treatment. In line with this, a number of survivors believed that the coaches “didn’t know anything about cancer treatments… didn’t have any medical knowledge” (CS4) or didn’t have “a full understanding” (CS2). However, others expressed that they did not expect that kind of knowledge from the coaches, noting that they “seemed knowledgeable enough” (CS5) and because “so many treatments and so many tablets are different, it’s almost impossible to have all the information” (CS3).

##### Gaps in family support

A need for emotional support to be provided to family members of cancer survivors was identified, particularly for the families of terminal patients. In the same way the support can cease for survivors once they have completed their cancer treatment, the support for family members is also seen to be lacking once a patient passes away. MindStep coaches remarked that patients “asked us if the families can do the program instead of them” (MSC3), as they were worried about their families and “how they might deal after I [patient] die” (MSC3). When patients had passed away, hospital staff recalled comments from their family members around a desire for more support; “They’ve said … ‘I went out into this kind of void and I didn’t know who to talk to, I was no longer coming to the hospital every day. I lost all this inbuilt support and I had not a clue what to do after that’” (HS1, recounting a family member’s comments).

## Quantitative results

Cancer survivors who completed the CSP in the 15-month pilot period (*N* = 25; 84% female; M age = 61 years; age range 32–84 years) had a range of cancer diagnoses (48% breast cancer, 24% lymphoma, 8% squamous cell carcinoma, and the remaining 20% with either colorectal, neuroendocrine, fallopian tube, tonsil, melanoma, or prostate cancer). On average, the cancer survivors had 6 phone calls (range = 4–9 phone calls) with a coach during the CSP and spent 172 days (range = 92–279 days) in the telephone-delivered components of the CSP. Outcomes of measures obtained are shown in Table 2. Physical activity increased from an average of 96 minutes at baseline to an average of 205 minutes per week at completion of the CSP. Patients entered the program with a mean activation of 66.5 points, or level three on the PAM scale, indicating they were beginning to engage in positive health and self-management behaviours [27]. The mean difference between baseline and post-CSP in patient activation was an improvement of 4.8 points, with improvements of 3 and 6 points over a 6-to-12-month period deemed as acceptable and excellent outcomes respectively, by the American College of Physicians [30]. At baseline, survivors presented with moderate unmet informational and emotional needs, and no-to-low unmet financial or access needs. Needs improved across the four domains, with informational and emotional needs changing from moderately unmet at baseline, to “needs met” post-CSP, on average.

**Table 2:** Pre- and post-Cancer Survivorship Program scores shown as mean (standard deviation) (*N* = 25)

	Weekly physical activity (minutes)	PAM-10 score*	Information needs**	Financial needs**	Access and continuity of care needs**	Coping, sharing, and emotional needs**
Pre-CSP	96 (90.2)	66.5 (12.8)	1.6 (0.8)	1.1 (1.0)	0.9 (0.6)	1.9 (0.9)
Post-CSP	205 (109.6)	71.3 (15.4)	0.8 (0.8)	0.5 (0.9)	0.5 (0.9)	0.8 (0.9)

*Higher scores indicate a more “activated”/ self-managed individual

** < 1.5 indicates no-to-low unmet needs, 1.5 to 3 indicates moderate unmet needs, and > 3 indicates high unmet needs

## Discussion

Cancer survivorship in Australia continues to increase due to new methods being used for early detection and advancements in treatment technologies [1, 6]. Beyond the active treatment phase, those who have survived cancer often contend with a number of ongoing health-related and social challenges as a result of the disease [6], which highlights a critical need for a continuum of care into the survivorship and self-management phase [12]. While some survivorship interventions have indicated the potential benefits of supporting cancer survivors in the post-treatment phase [12, 15], cancer survivors have generally reported feeling unsupported in the survivorship phase of their cancer journey [13].

The current study attempted to address this knowledge and practice gap. A telephone-delivered cancer survivorship program (CSP) was developed and piloted in Eastern Australia in the private health care setting. Using a mixed methods approach, the current study explored cancer survivors’, coaches’, and hospital staff experiences of the Ramsay Connect Cancer Survivorship Program through semi-structured interviews. Cancer survivor outcomes were also described with relevant quantitative data, routinely collected throughout the program.

While the quantitative analysis was limited to descriptive statistics with a small sample, the preliminary data from this pilot indicated that the CSP may have been beneficial for cancer survivors across a number of relevant outcomes. Participating cancer survivors appeared to be more physically active, highly “activated”, and their unmet needs had been reduced across all domains post-program. The findings pertaining to physical activity were particularly promising, given physical activity has the potential to improve cancer survivors’ quality of life, cancer survival, and management of cancer-related fatigue and pain [31].

The qualitative component of the research, which involved 17 interviews with cancer survivors, coaches, and hospital staff, provided in-depth perspectives of the CSP, highlighting where the program was successful and where improvements could be made. Three syntheses and 11 themes were generated through thematic analysis. The first synthesis centred around operational factors and highlighted challenges with recruitment and eligibility. Hospital staff expressed practical constraints in the clinical setting which made completing the initial paperwork and “after treatment care plan” (survivorship plan) a challenge. In US settings, nurses and physicians have acknowledged the value of survivorship plans, but have also voiced issues around staffing levels, busy patient loads, and other responsibilities limiting their ability to incorporate this into their practices [32]. In the same US study, all participants felt strongly that physicians needed to reinforce the importance of the nursing role with cancer patients [32]; this also emerged in the current study, with hospital staff stating that patients would take a physicians advice to join the CSP, over a nurse’s suggestion.

Eligibility for the CSP was another central point of discussion with participants, and the need to appropriately time and tailor the program emerged as critical. Hospital staff originally found it difficult to identify cancer survivors meeting the CSP eligibility criteria, which required them to be finishing active treatment. In line with a recent meta-review by Laidsaar-Powell et al. [33], the hospital staff commented that there are few cancer treatments now which are straightforward. Similarly, qualitative research with cancer patients in the UK found that the majority of participants were still receiving some form of treatment, while only a minority were going off of treatment [15]. In the current study this barrier meant that, despite being a program intended for post-treatment phase, the program was opened to cancer survivors in ongoing treatment. While participants believed that the program support could be beneficial at any stage of the cancer journey, it was emphasised that it would need to be appropriately tailored to do so. Participants suggested that program participation early in their cancer journey may have been too much commitment, which was also echoed by cancer patients in the UK, who described being offered support too early in their cancer journey, but feeling unsupported when future needs occurred [15].

Another key theme generated focused on connection across the care team in the CSP. While some links were built between hospital cancer care navigators and health coaches in the referral process, connection between members of the broader multidisciplinary care team was seen to be lacking across the program. The main barriers to this connection were described as time management and patient confidentiality issues. While the CSP focused on integration between hospital cancer care navigators and health coaches, it was not designed for a greater level of integration with the broader multidisciplinary team (e.g. specialist doctors) in this pilot. Two studies by Walsh and colleagues noted the same barriers experienced by health professionals in other Australian cancer healthcare settings [34, 35]. Specifically, ineffective information exchange, inadequate communication between specialist and primary care, managing scarce resources, time constraints, and difficulties arranging meetings, were cited as the greatest challenges to effective care team communication.

In a systematic review and meta-analysis of 30 years of cancer research, Gorin and colleagues suggested that increased communication across multidisciplinary teams could improve cancer coordination [36]. They reported that coordinated approaches to cancer care were almost twice as efficacious (OR = 1.9, 95% CI = 1.5–3.5) in improving appropriate use of healthcare services, over care as usual. Across the literature, a coordinated approach to cancer care led to improvements in 81% of outcomes, such as patient experiences and quality of life. In research focussing on prostate cancer survivorship specifically, a fragmented approach to care was almost three times as costly as a coordinated approach with an ongoing survivorship care team. These findings indicate the importance of ensuring communication across members of the care team, and a need to address current barriers in achieving this, to ensure a coordinated care response, particularly in the survivorship phase [37].

The second synthesis indicated that the CSP focused on appropriate information which addressed the challenges survivors faced following cancer treatments. Previous research with clinicians has highlighted the importance of evaluating survivors’ needs in the transition to the chronic phase of cancer management, while survivors themselves have also expressed a need for guidance to improve their symptom management in the post-treatment phase [15]. The CSP was able to address both practice gaps, by assessing survivors’ unmet needs and having health coaches guide symptom management as an integral part of the program.

Importantly, all participants interviewed in the current study noted that the CSP filled a gap in support that survivors tend to experience in the transition from active treatment to the home/community context. This gap has been well-documented in the literature, with cancer survivors commonly voicing that they do not know what to expect once active care is over, and some reporting that they felt uncared for at this point in their cancer journey [32]. According to Walsh and colleagues [34], it is a widely held view among survivors, clinicians, and carers that the follow-up care required for cancer survivors can be complex and overwhelming. A need for administrative support and arranging appointments has been voiced [34], as survivors often have a poor understanding of the services and support pathways available to them for issues occurring when they are no longer receiving active treatment [15, 35]. Currently, in Australia there is no broad mechanism for ongoing care. For example, in the private setting where the current study was conducted, once treatment ends and the patient is not admitted to hospital, the hospital is no longer funded by the private health insurer. These funding models limit the health care providers’ ability to provide ongoing support.

Cancer survivors in the current study, as well as previous qualitative work by Harley et al. [15], expressed a real desire to get back to normal life. The CSP worked towards this by empowering survivors to self-manage their health, which was considered an effective approach by survivors, coaches, and hospital staff. Cancer care coordinators in other settings have also expressed the importance of helping empower patients to self-manage the challenges of their disease [34], affirming the approach taken in the CSP.

A number of survivors and coaches in the current study described the psychological difficulties individuals experienced in the survivorship phase of their cancer journey. Consistent with feedback from cancer patients in other studies, survivors in the current study expressed fears around cancer recurrence, stresses associated with upcoming scans, and other generalised psychological distress as a result of the cancer journey [15, 31, 38, 39]. While cancer survivors in other studies have expressed feeling satisfied with their physical health care, they have also noted that their psychological needs were not met, and they have generally agreed that counselling should be a part of follow-up care [32, 40]. Despite this, hospital staff in the current study described patient hesitancy to seek out and uptake support through psychological services, mainly due to social stigmas and associated costs. Survivors themselves also spoke of concerns about burdening or worrying their families with their mental health difficulties. These sentiments around stigma and family burden echo feedback from cancer survivors in other settings [15].

The findings emphasised that just having somebody in their corner, away from the family, was viewed as the most beneficial aspect of the CSP. This is consistent with feedback from previous Australian research [34], in which cancer patients and clinicians considered the allocation of a ‘key contact’ person to be essential for patient advocacy, care coordination, and a sense of ongoing support and advice during treatment. Similarly, in an Australian national survey of women with breast cancer, 42% of participants reported that they would have liked to have a main contact person to go to (41). The CSP uniquely addressed this gap by providing survivors with a health coach who was in their corner.

In the CSP, survivors who were identified as experiencing psychological distress through routine screening, had the option of being referred to MindStep, a low-intensity cognitive behavioural therapy program. However, a number of cancer survivors declined MindStep, preferring to just stay with their health coach after rapport had been built. This suggests that all coaches (or ‘key contacts’) may require training and upskilling to be able to provide mental health support to cancer survivors, even if this is not their primary role. In particular, flexible counselling skills which allow survivors to talk about their journey and feel heard were described by the MindStep coaches as necessary when working with this cohort.

### Limitations

The findings from this study must be considered while appreciating some limitations. Firstly, the timeline of interviews may have affected participant feedback. For example, some survivors were interviewed several months after they had completed the program, meaning their ability to recall specific details about their experiences was lacking in some instances. Similarly, the timing of interviews with the health coaches and MindStep coaches also impacted their ability to share their experiences of the CSP. Given the CSP was still relatively new, some coaches reported working with few cancer survivors at the point of interview, which limited the breadth and depth of their feedback. It must also be acknowledged that the coaches were employed by Remedy Healthcare, while the hospital staff were employed by Ramsay Healthcare Australia; these organisations set up the CSP and funded this research, meaning these participants could have possibly been reserved or biased in their responses. To mitigate this limitation, care was taken to ensure the interviews were conducted and the data were handled and de-identified by the Flinders researchers only, so that participants were guaranteed anonymity in their feedback. Finally, given this study presents results from the pilot phase of the CSP, the sample available was small and lacked a control group. As such, only descriptive statistics of survivor outcomes could be presented, with a lack of statistical power for sophisticated analyses and significance testing. Although preliminary outcomes in physical activity, patient activation, and unmet needs were promising, caution must be taken when interpreting these quantitative outcomes.

## Conclusion

Cancer survivors face a range of challenges in the survivorship phase and require a level of ongoing support post-active treatment. This telephone-delivered CSP piloted in Australia, which utilised coaches, appeared to be beneficial to cancer survivors. Preliminary data indicated improvements in physical activity, activation level, and unmet needs across the program, although further large-scale quantitative evaluation is required. Qualitative feedback highlighted that survivors appreciated the support offered through the CSP, and the module content, focus on empowerment, and mental health support were especially valued. Just *having somebody in their corner* was viewed as central to the cancer survivor experience of the CSP.

A number of operational challenges, particularly around eligibility, recruitment, and follow-up were highlighted by hospital staff, which emphasised a need to streamline communication and consider the demands experienced in clinical settings. The biggest difficulty health coaches faced was around providing mental health support to cancer survivors, as well as understanding the complexities of cancer types and treatments. While components of the CSP were believed to be of benefit to individuals at various stages of their cancer journey, it was emphasised that the program would need to be tailored appropriately, with clear parameters and expectations, to achieve this.

## Data Availability

Data are not available to share due to ethics approval restrictions.

## References

[1] Australian Institute of Health and Welfare (2019) Cancer in Australia 2019. Cancer series no.119. Cat. no. CAN 123. AIHW, Canberra

[2] Bates N, Callander E, Lindsay D, Watt K (2018). CancerCostMod: a model of the healthcare expenditure, patient resource use, and patient co-payment costs for Australian cancer patients. Heal Econ Rev.

[3] Muñoz RDMSNNPCPHNO, Farshidpour LBS, Chaudhary UBMD, Fathi AHMD (2018). Multidisciplinary cancer care model. Clin J Oncol Nurs.

[4] Levit L, Balogh E, Nass S, Ganz PA (2013). Patient-centered communication and shared decision making. Delivering high-quality cancer care: charting a new course for a system in crisis.

[5] Tighe M, Molassiotis A, Morris J, Richardson J (2011). Coping, meaning and symptom experience: a narrative approach to the overwhelming impacts of breast cancer in the first year following diagnosis. Eur J Oncol Nurs.

[6] Shapiro CL (2018). Cancer survivorship. N Engl J Med.

[7] Espie CA, Fleming L, Cassidy J, Samuel L, Taylor LM, White CA (2008). Randomized controlled clinical effectiveness trial of cognitive behavior therapy compared with treatment as usual for persistent insomnia in patients with cancer. J Clin Oncol.

[8] Bower JE, Ganz PA, Desmond KA, Rowland JH, Meyerowitz BE, Belin TR (2000). Fatigue in breast cancer survivors: occurrence, correlates, and impact on quality of life. J Clin Oncol.

[9] Gielissen MF, Verhagen S, Witjes F, Bleijenberg G (2006). Effects of cognitive behavior therapy in severely fatigued disease-free cancer patients compared with patients waiting for cognitive behavior therapy: a randomized controlled trial. J Clin Oncol.

[10] Kroenke K, Spitzer RL, Williams JB, Löwe B (2010). The patient health questionnaire somatic, anxiety, and depressive symptom scales: a systematic review. Gen Hosp Psychiatry.

[11] Zebrack BJ (2011). Psychological, social, and behavioral issues for young adults with cancer. Cancer.

[12] Kvale EA, Huang CHS, Meneses KM, Demark-Wahnefried W, Bae S, Azuero CB (2016). Patient-centered support in the survivorship care transition: outcomes from the Patient-Owned Survivorship Care Plan Intervention. Cancer.

[13] Lubberding S, van Uden-Kraan CF, Te Velde EA, Cuijpers P, Leemans CR, Verdonck-de Leeuw IM (2015). Improving access to supportive cancer care through an e H ealth application: a qualitative needs assessment among cancer survivors. J Clin Nurs.

[14] Fitch M, Zomer S, Lockwood G, Louzado C, Shaw Moxam R, Rahal R (2019). Experiences of adult cancer survivors in transitions. Support Care Cancer.

[15] Harley C, Pini S, Bartlett YK, Velikova G (2012). Defining chronic cancer: patient experiences and self-management needs. BMJ Support Palliat Care.

[16] Grunfeld E, Levine MN, Julian JA, Pond GR, Maunsell E, Folkes A (2011). Results of a multicenter randomized trial to evaluate a survivorship care plan for breast cancer survivors. J Clin Oncol.

[17] Vardy JL, Chan RJ, Koczwara B, Lisy K, Cohn RJ, Joske D (2019). Clinical Oncology Society of Australia position statement on cancer survivorship care. Aust J Gen Pract.

[18] Chan RJ, Crichton M, Crawford-Williams F, Agbejule OA, Yu K, Hart NH (2021). The efficacy, challenges, and facilitators of telemedicine in post-treatment cancer survivorship care: an overview of systematic reviews. Ann Oncol.

[19] Pham Q, Hearn J, Gao B, Brown I, Hamilton RJ, Berlin A (2020). Virtual care models for cancer survivorship. NPJ Digit Med.

[20] Hunter J, Smith C, Delaney GP, Templeman K, Grant S, Ussher JM (2019). Coverage of cancer services in Australia and providers’ views on service gaps: findings from a national cross-sectional survey. BMC Cancer.

[21] Goldsbury DE, Yap S, Weber MF, Veerman L, Rankin N, Banks E (2018). Health services costs for cancer care in Australia: estimates from the 45 and Up Study. PLoS ONE.

[22] Rana RH, Alam K, Gow J, Ralph N (2019). Predictors of health care use in Australian cancer patients. Cancer Manag Res.

[23] Venning A, Oswald TK, Barnes M, Glover F, Lawn S, Azadi L (2021). It’s what’s under the hood that counts: comparing therapeutic outcomes when using Australian versus UK-produced clinical materials in an Australian mental health program. Aust Health Rev.

[24] Lawn S, Huang N, Zabeen S, Smith D, Battersby M, Redpath P (2019). Outcomes of telephone-delivered low-intensity cognitive behaviour therapy (LiCBT) to community dwelling Australians with a recent hospital admission due to depression or anxiety: MindStep™. BMC Psychiatry.

[25] Braun V, Clarke V (2006). Using thematic analysis in psychology. Qual Res Psychol.

[26] Campbell HS, Hall AE, Sanson-Fisher RW, Barker D, Turner D, Taylor-Brown J (2014). Development and validation of the Short-Form Survivor Unmet Needs Survey (SF-SUNS). Support Care Cancer.

[27] Hibbard JH, Stockard J, Mahoney ER, Tusler M (2004). Development of the Patient Activation Measure (PAM): conceptualizing and measuring activation in patients and consumers. Health Serv Res.

[28] Rademakers J, Nijman J, van der Hoek L, Heijmans M, Rijken M (2012). Measuring patient activation in the Netherlands: translation and validation of the American short form Patient Activation Measure (PAM13). BMC Public Health.

[29] Hibbard JH, Greene J (2013). What the evidence shows about patient activation: better health outcomes and care experiences; fewer data on costs. Health Aff.

[30] American College of Physicians (2022) Gains in patient activation (PAM) scores at 12 months [Available from: https://www.acponline.org/clinical-information/performance-measures/gains-in-patient-activation-pam-scores-at-12-months]. Accessed 14 Nov 2022

[31] Emery J, Butow P, Lai-Kwon J, Nekhlyudov L, Rynderman M, Jefford M (2022). Management of common clinical problems experienced by survivors of cancer. Lancet.

[32] Hewitt ME, Bamundo A, Day R, Harvey C (2007). Perspectives on post-treatment cancer care: qualitative research with survivors, nurses, and physicians. J Clin Oncol.

[33] Laidsaar-Powell R, Konings S, Rankin N, Koczwara B, Kemp E, Mazariego C (2019). A meta-review of qualitative research on adult cancer survivors: current strengths and evidence gaps. J Cancer Surviv.

[34] Walsh J, Young JM, Harrison JD, Butow PN, Solomon MJ, Masya L (2011). What is important in cancer care coordination? A qualitative investigation. Eur J Cancer Care.

[35] Walsh J, Harrison JD, Young JM, Butow PN, Solomon MJ, Masya L (2010). What are the current barriers to effective cancer care coordination? A qualitative study. BMC Health Serv Res.

[36] Gorin SS, Haggstrom D, Han PKJ, Fairfield KM, Krebs P, Clauser SB (2017). Cancer care coordination: a systematic review and meta-analysis of over 30 years of empirical studies. Ann Behav Med.

[37] Zhao Y, Brettle A, Qiu L (2018). The effectiveness of shared care in cancer survivors-a systematic review. Int Journal Integrated Care.

[38] Keesing S, McNamara B, Rosenwax L (2015). Cancer survivors’ experiences of using survivorship care plans: a systematic review of qualitative studies. J Cancer Surviv.

[39] Chua GP, Ng QS, Tan HK, Ong WS (2021). Cancer survivors: what are their concerns and quality of life across the survivorship trajectory?. J Cancer Sci Clin Ther.

[40] Khan N, Evans J, Rose P (2011). A qualitative study of unmet needs and interactions with primary care among cancer survivors. Br J Cancer.

[41] Williams P, Rankin N, Redman S, Davis C, Armstrong B, Malycha P (2004). National survey of women with early breast cancer: their perceptions of care.

